# Carbon Monoxide Poisoning and Flooding: Changes in Risk Before, During and After Flooding Require Appropriate Public Health Interventions

**DOI:** 10.1371/currents.dis.2b2eb9e15f9b982784938803584487f1

**Published:** 2014-07-03

**Authors:** Thomas Waite, Virginia Murray, David Baker

**Affiliations:** Specialty Registrar in Public Health and ToxicologyPublic Health England; Extreme Events and Health Protection, Public Health England, London, UK; Extreme Events and Health Protection, Public Health England, London, UK

## Abstract

Introduction While many of the acute risks posed by flooding and other disasters are well characterised, the burden of carbon monoxide (CO) poisoning and the wide range of ways in which this avoidable poisoning can occur around flooding episodes is poorly understood, particularly in Europe. The risk to health from CO may continue over extended periods of time after flooding and different stages of disaster impact and recovery are associated with different hazards. Methods A review of the literature was undertaken to describe the changing risk of CO poisoning throughout flooding/disaster situations. The key objectives were to identify published reports of flood-related carbon monoxide incidents that have resulted in a public health impact and to categorise these according to Noji’s Framework of Disaster Phases (Noji 1997); to summarise and review carbon monoxide incidents in Europe associated with flooding in order to understand the burden of CO poisoning associated with flooding and power outages; and to summarise those strategies in Europe which aim to prevent CO poisoning that have been published and/or evaluated. The review identified 23 papers which met its criteria. The team also reviewed and discussed relevant government and non-government guidance documents. This paper presents a summary of the outcomes and recommendations from this review of the literature. Results Papers describing poisonings can be considered in terms of the appliance/source of CO or the circumstances leading to poisoning.The specific circumstances identified which lead to CO poisoning during flooding and other disasters vary according to disaster phase. Three key situations were identified in which flooding can lead to CO poisoning; pre-disaster, emergency/recovery phase and post-recovery/delayed phase. These circumstances are described in detail with case studies. This classification of situations is important as different public health messages are more appropriate at different phases of a disaster. The burden of disease from poisoning caused by each potential source and at each phase of a disaster is different. CO poisoning is not compulsory and deaths associated with a flood but delayed for a period of months, for example due to a damaged boiler, may never be attributed to the flood as surveillance often ends once the floodwaters recede. The problem of under–reporting is crucial to our understanding of flooding-related poisoning. The indoor use of portable generators, cooking and heating appliances designed for use outdoors during periods of loss of mains power or gas is a particular problem. In the recovery phase, equipment for pumping, dehumidifying and drying out of properties poses a new risk. In the long term, mortality and morbidity associated with the renewed use of boilers which may have suffered covert damage in flooding is recognised but very difficult to quantify. Papers evaluating interventions were not found and where literature exists on prevention of CO poisoning in disaster situations, it is from the USA. Conclusions This paper for the first time describes the different risks of CO poisoning posed by the different phases of a disaster. There is a specific need to recognise that any room in a building can harbour a CO emitting appliance in flooding; wood burners and rarely used chimney flues may become particularly problematic following a flood. Recommendations 1) Public health workers and policy makers should consider establishing toolkits using the CDC toolkit approach; the acceptability of any intervention must be evaluated further to guide informed policy. 2) CO poisoning must form part of syndromic and event based surveillance systems for flooding and should be included in measures of the health impact of flooding. 3) CO monitors in the domestic environment should be sited not only in proximity to known CO emitters but also in locations where mobile or short term CO emitting appliances may be placed, including woodburners and infrequently used fireplaces.

## Introduction

Flooding occurred in 50 of the 53 countries in the WHO European Region during the past decade, with the most severe floods in Romania, the Russian Federation, Turkey and the United Kingdom.^1^ Across the UK, Over 6,500 homes were flooded in winter 2013/14; many thousands were affected by disruption to infrastructure and essential services, including energy supplies. Such disruption lasted for weeks or even months in some areas. River flooding is projected to affect 250 000–400 000 additional people per year in Europe by the 2080s, more than doubling the numbers from those in 1961–1990. The populations most severely affected will be those of central Europe and the British Isles^1^^,^ 2.

Carbon monoxide (chemical formula CO) is a poisonous gas which is produced when any carbon-containing fuel, such as hydrocarbon gas, petrol, oil or wood, combusts incompletely. When CO is inhaled, the gas binds to haemoglobin in red blood cells, reducing the ability to carry oxygen and thus starving vital organs of oxygen. CO is odourless and colourless; it is therefore undetectable by humans without the use of specialist detection equipment.

The effects of poisoning vary according to dose; as the percentage of CO in the blood rises, symptoms worsen. Shortness of breath and headache occur at lower doses, followed by dizziness, loss of vision, confusion, collapse, coma, convulsions and ultimately brain damage or death. Long term effects are also well described such as memory impairment or problems with concentration or attention.

Carbon monoxide poisoning is a major public health problem in Europe. The annual death rate in Europe is 2.2 people per 100,000 population which is similar to the death rate from HIV/AIDS in 2010 at 2.0 per 100,000 or skin cancer at 2.1 per 100,000^3^ . However it is difficult to quantify the health harm and deaths caused by CO due to the complexity of detecting and reporting poisonings. Official UK data^4^ state that there are around 40 deaths per year from CO poisoning in England and Wales. A recent examination of data held by 28 European Union (EU) states found that over 140,000 deaths had been caused by CO over a 28 year period of which over half were accidental^3^. The true level of death and disability caused by CO is probably higher still as exposure is often under -diagnosed or misdiagnosed due the often non–specific nature of the symptoms caused^5^^,^ 6. CO poisoning has been reported to be a particular problem in the aftermath of a disaster as people engage in more high-risk behaviours^7^^,^ 8. In particular, flooding, power cuts and post-disaster cleanup and recovery are primarily associated with both fatal and non-fatal disaster-related CO poisonings^9^. For example, in a review of disaster-related poisonings, Iqbal et al 10 found that 89% of fatal CO poisonings occurred within 3 days of the onset of disaster. It is not currently possible to establish how many cases of poisoning are attributable to flooding or other extreme events although some EU Member States, including France, have established surveillance systems^11^. A 2004 literature review of the public health impacts of floods and chemical contamination found few relevant publications overall 12; of the three identified reports on ‘naturally occurring’ floods, two considered the problems of associated carbon monoxide poisoning.

In this paper we therefore describe from a literature review the different and changing situations in which people may be poisoned by CO following a flood. We use the Framework of Disaster Phases^13^ in order to enable policy makers and disaster workers to provide tailored advice as a disaster evolves and the affected community recovers.

## Method

A literature review was undertaken with three key objectives:

- To identify published reports of flood-related carbon monoxide incidents that have resulted in a public health impact and to categorise these according to Noji’s Framework of Disaster Phases^13^.
- To summarise and review carbon monoxide incidents associated with flooding in order to understand the burden of CO poisoning associated with flooding and power outages
- To summarise those strategies which aim to prevent CO poisoning that have been published and/or evaluated

**Search strategy**

The SafetyLit, Embase and Medline databases were searched without time or language restrictions. SafetyLit is an Injury Research and Prevention Literature Database and is a service of the World Health Organisation and San Diego State University. It covers approximately 14,000 scientific journals from 143 countries using defined sub-sections, including poisoning. The Cochrane Database, NHS EED and DARE were searched for systematic reviews; none were found.

The search was conducted in two phases. In the first phase, a title scan was performed to screen out irrelevant papers and duplicates. Abstracts were obtained for the second phase in which papers for inclusion in this review were selected. The chosen specific search terms were: monoxide; flood*; health. Only carbon monoxide incidents as a result of disasters (including flooding) in the English language were included.

**Analysis**

Full text articles were scrutinised for details of circumstances and appliances associated with carbon monoxide poisoning, in the context of any disaster setting. Where details of the circumstances of poisoning were described, these were classified according to the Disaster Phases Framework^13^.

Noji’s disaster phases framework allows disaster risks and injury prevention interventions to be considered in five distinct phases; Interdisaster phase, Predisaster/Warning phase, Impact phase, Emergency phase and Reconstruction Phase. For an overview of these phases, see figure 1. For the purposes of this paper, we have split the reconstruction phase into two chronological phases: ‘recovery phase’ and ‘late recovery phase’.

**The framework of disaster phases, circumstances which present a health risk from CO and public health actions to mitigate that risk (adapted from Noji’s 1997 Framework of disaster processes):**
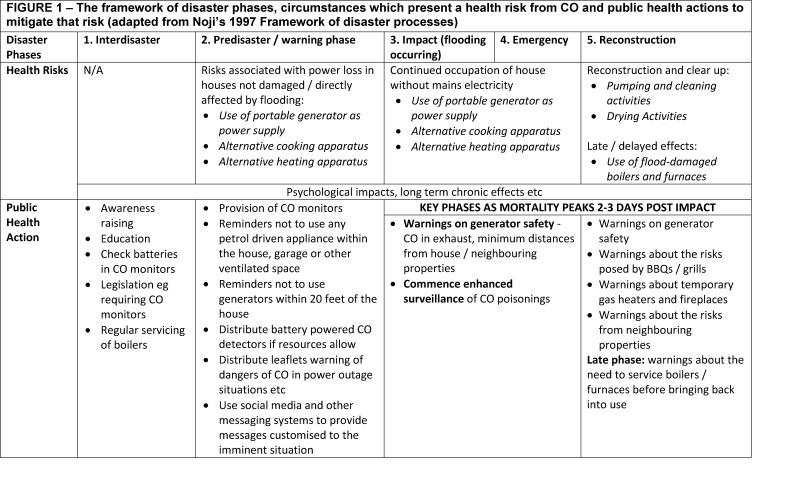

## Results

A total of 23 papers were included for review after title and abstract screening. All but two of these described disasters and floods in North America.

**Previous reviews**

A 2012 systematic literature review was identified, which provided an overview of surveillance and epidemiology of disaster-related CO poisoning in the United States only 10. 4% of the papers found in that review related to flooding. However many of the others highlighted the dangers associated with power cuts and thus can clearly be extrapolated to many disaster situations.

Iqbal et al reviewed 28 papers which described 362 incidents, finding that 88% of fatal CO poisonings reported to be associated with a disaster and 53% of non-fatal poisonings occurred within 3 days of the disaster. A two week period accounted for all reported fatalities and 97% of nonfatal cases. This striking difference temporal distribution of fatal and non-fatal CO poisonings points to differing risks occurring at different points in the aftermath of a flood and supports the need to consider different intervention strategies as a disaster progresses.

**Disaster Phases**

Papers describing poisonings can be considered in terms of appliance/source of CO or circumstances leading to poisoning. Appliances which have been associated with CO poisoning are illustrated in figure 2.

**Appliances which have been linked to Carbon Monoxide poisoning in disasters:**
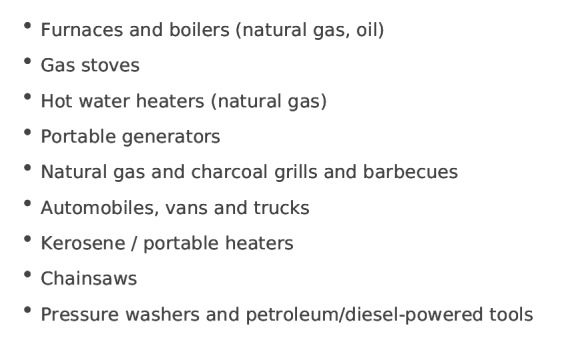
Adapted from CDC (2012). Original article uses “gas” for petroleum, defined in the USA here: http://www.eia.gov/petroleum/workshop/ngl/pdf/definitions061413.pdf

**Circumstances leading to poisoning**

The specific circumstances identified which lead to CO poisoning during flooding and other disasters vary according to disaster phase. Three key situations were identified in which flooding can lead to CO poisoning which can be considered to follow the chronology of the Framework of Disaster Phases 13 displayed in figure 1. This classification is important as different public health messages are clearly more appropriate at different phases of a disaster. The circumstances are summarised below and described in more detail with case studies later:

- **Pre disaster / Impact**
  - Use of portable generators as power supply
  - Cooking
  - Heating / warmth
  - Lighting

- **Emergency / recovery phase**
  - Pumping and cleaning activities
  - Dehumidifying and drying activities

- **Post recovery / delayed phase**
  - Use of flood damaged boilers / furnaces

Papers evaluating interventions were not found and where literature exists on prevention of CO poisoning in disaster situations, it is from the USA.

The burden of disease from poisoning caused by each potential source and at each phase of a disaster is different. However, reporting of CO poisoning is not compulsory and deaths associated with a flood but delayed for a period of months, for example due to a damaged boiler, may never be attributed to the flood as surveillance often ends once the floodwaters recede.

## Key findings

The problem of under–reporting is crucial to our understanding of flooding-related poisoning. Not all flooding related CO poisonings and deaths are recognised as such and even fewer are published in journals. Iqbal et al have summarised the reasons for the underestimation of the total number of disaster related CO deaths^10^. For similar reasons, the true number and variety of appliances and circumstances in which people have been poisoned by CO associated with flooding is almost certainly under - represented in scientific literature.

**Mains power cuts/outage during (and after) flooding**

The loss of mains electricity, even in the absence of overt flooding of a property, is a major contributor to carbon monoxide poisoning. Power outages can affect a large area; within that area, some houses will be flooded whilst others may not be. Some houses may remain inhabitable even after minor flooding. The context of continued dwelling in a house without power presents a number of risks from CO poisoning from various domestic activities (see following sub-sections).

Use of portable generators as alternative electricity / power supply

The CO source responsible for the greatest number of poisonings in disaster situations is the portable generator, accounting for up to 76% of fatalities^10^. The exhaust from a 5.5kW generator will generate the same amount of CO as six cars, which quickly and easily builds up to fatal levels in any indoor environment^14^. 20 of 30 incidents found by Iqbal et al involving generators were due to the use of generators indoors. 33% of cases stemmed from the placement of generators in garages, close to windows or outdoor (including air conditioning) vents. In one study of the incidence and mechanisms of carbon monoxide poisoning during the first five days after Hurricane Rita 15, improper placement of portable generators in indoor locations or close to air conditioning intake vents was responsible for all 21 CO exposures seen at one hospital, resulting in 5 fatalities, 1 brain death and 13 people requiring short term treatment.

In some circumstances, access to power for lighting and electricity is necessary for medical equipment, such as dialysis machines and powered oxygen supplies 16. It is important to note that, despite advice to evacuate or leave a home which is thought to be at risk from flooding or power outages, some people choose to stay in their own homes. This group may be considered to be particularly at risk, but also a target group for advice or interventions, such as the provision of information or CO monitors. Portable generators represent a predictable risk but one which is inadequately appreciated by the public.

Cooking

Houses which are usually dependent on electricity for cooking will find themselves unable to cook food during any interruption to the power supply. Iqbal et al found the indoor use of outdoor barbecues and charcoal grills in this situation to be the second most common source of CO poisoning^10^. Inappropriate use of a grill or BBQ was thought to be the CO source in nearly a quarter of all incidents, a burden which changes according to the nature or type of the disaster. CO poisoning following the use of grills and BBQs indoors has notably been described in association with a severe ice storm in Canada^17^. With the recent popularity of “smokehouse” restaurants in the UK^18^, a wider population may now mistakenly consider this sort of BBQ cookery indoors to be safe and resort to it in the event of a disaster; for example, the use of bottled milk to feed babies requires access to boiled water to sterilise bottles. There is also a considerable fire risk from the use of such appliances. The risk of poisoning from indoor barbecues was identified in the UK in a study which analysed sources after CO detectors alarmed in homes^19^. This entirely avoidable risk of CO poisoning merits urgent public health work as the indoor use of solid fuel stoves and cooking appliances has been associated in several studies with particular racial, ethnic or cultural groups^9^^,^ 10 ^,^ 19 ^,^ 20 ^,^ 21.

Heating

Gas and solid fires have long been recognised as a potential source of carbon monoxide exposure, even in the absence of a disaster. There are many types of these appliances in use across Europe, ranging from fixed gas or coal fireplaces to wood burners and portable gas stoves. There are many case reports associated with the use of such appliances; it is perhaps unsurprising that in the absence of mains power, families or individuals in cold houses will turn to rarely used or poorly maintained appliances to provide extra heat. Iqbal found that in a quarter of all disaster-related nonfatal CO poisoning cases, the most likely source was a heater or stove^10^. There have also been many reports of fires associated with the use of infrequently used appliances, including chimney fires associated with poorly maintained fireplaces and blocked flues. The importance of routine maintenance of all gas appliances and solid fuel burners including chimneys must be reinforced as part of routine public safety advice, with specific mention made of appliances which will be of use in an emergency.

Recovery phase (acute) – pumping out and cleaning activities

The recovery phase after flooding is associated with different carbon monoxide hazards than during the disaster itself, these stem from clean up and recovery activities. The first of these, chronologically, is the risk of CO poisoning during efforts to pump out flood waters and clean up affected areas. A variety of high - risk equipment may be used, particularly pressure washers and pumping equipment with built in generators. All of these require power, which is generally in the form of petrol driven generators, either stand - alone or built into the equipment.

In 1997, the town of Grand Forks, North Dakota (USA) was hit by severe flooding. In the aftermath, 33 laboratory confirmed cases of CO poisoning were identified in 18 separate incidents. Every case was from the use of petrol-powered equipment to wash and clean out flooded basement areas. Five incidents, affecting 16 people, were from professional cleaners, the remainder being members of the public. 30 people had considered the risks from generators but inadequately characterised that risk as they thought the basement area was sufficiently ventilated prior to using the equipment^22^.

Since basement areas are among the first parts of the home to be flooded, the use of such equipment in these enclosed, often below ground areas must be considered to be particularly high risk behaviour. CDC advice is clear that there are no circumstances in which it is safe to use a petrol or diesel driven generator in an indoor environment, or less than 20 feet from the nearest door, window or vent 23.

Recovery phase (acute) – dehumidifying and drying activities

Following the removal of overt flood water and associated mud and debris, the next step in the recovery process is dehumidifying and drying a property. The problems of an absence of mains power persist even after the removal of water as mains power supplies may be damaged or destroyed within affected properties or further up the distribution grid / infrastructure supplying those dwellings.

Appliances used to dry out properties may include portable heat sources such as portable gas (butane or propane) fuelled heaters as well as electrical equipment such as dehumidifiers attached to portable petrol or diesel-driven generators. The risks associated with equipment such as barbecues and other solid fuel appliances have already been discussed.

This hazard is not peculiar to the domestic environment. Following flooding in England in 2008, a father and son died after exposure to CO emitted by a generator being used as they were pumping out the cellars of a community rugby club in Tewkesbury in 2007 24 .

Post-flooding (delayed / chronic) – using flood damaged domestic appliances including boilers/furnaces

The late or delayed burden of disease from CO poisoning occurs after recovery and reoccupation of property and is associated with the use of mains gas or oil boilers or furnaces for central heating and provision of hot water.

Flood water causes damage to domestic boilers/furnaces used to heat water for central heating as well as washing and other domestic functions. Most of these systems burn gas or oil to produce heat requiring a flue for the extraction of noxious combustion products including carbon monoxide. It is recommended that such appliances should be serviced on an annual basis. Flooding can increase the risk of CO poisoning from these appliances in two ways, causing direct damage to the appliance which affects the combustion process or by causing debris to obstruct the flue. It is therefore essential to have a boiler or furnace serviced following flooding before switching it back on 25.

This burden of delayed morbidity (and mortality) is most likely to be under-reported; disaster health surveillance may be geographically and temporally restricted to only the time period for which flooding existed. People may be displaced from their home for months or years following flood damage; approximately 33% of households affected by the 2007 flooding in England were still unable to return home in May 2008^26^. CO poisoning as a secondary health consequence of flooding is thus easily overlooked. Furthermore, if flooding occurs in the summer, it is conceivable that the risk from a faulty boiler may not become a hazard until many months later in the cold weather of the following winter, when the appliance is brought back into regular use. In this way, the association between flooding and CO poisoning may not be made. This problem of under - reporting is compounded by the fact that not all accounts of disaster related CO poisoning are published, even where they are recognised as being associated with a particular incident^10^.

**Interventions**

Limited literature exists on the prevention of CO poisoning in any disaster situation, including flooding. In general, such publications are from the USA. In the UK, Public Health England has published recovery handbooks for chemical, biological and radiation incidents. None of these address the potential scenario of widespread carbon monoxide poisoning in power failures. A similar recovery handbook for extreme weather events is planned but does not yet exist. Prevention of CO poisoning will be an important part of any disaster recovery plan.

Public education about the risks of CO poisoning is a major component of injury prevention, including poisonings. Education and awareness encompasses a wide spectrum of activities; some can be considered to raise general awareness, whilst others form a reactive response to an acute problem. The United States CDC carbon monoxide prevention toolkit has elements of both of these approaches and covers both emergency (disaster) situations and non-emergency messaging. The toolkit summarises the most common scenarios for CO poisoning, identifies at risk population and describes behaviours that put people at risk. Furthermore, it highlights ‘audience-tested’ awareness and prevention messages and contains customisable materials that can be adapted at state level to create a prevention campaign. No similar resource was found from Europe. We have categorised the suggested interventions according to the Framework of Disaster Phases; these are described at figure 1.

Increasing awareness of the risk of CO poisoning amongst social and health care workers is also important, given the non-specific nature of the symptoms of early CO poisoning. In winter 2013, the UK CMO published a letter to all healthcare workers which, as well as seeking to raise awareness of the dangers of CO poisoning, provides a flowchart to aid diagnosis using the “COMA” tool supported by the RCGP and College of Emergency Medicine^27^^,^ 28.

Public health interventions are typically evaluated based on estimates of the lives saved (premature deaths avoided) and other criteria such as acceptability or reduction of health inequalities 29 . Whilst it is probable that a variety of strategies and public awareness interventions to reduce CO poisonings in disaster situations have been undertaken around the world, there is very little published information on formal assessments (qualitative or quantitative) of the effectiveness of the awareness campaigns as a whole or of individual intervention measures. Campaigns (national or local) will have different impacts on different communities and populations; it is rarely possible to directly compare the impact of communications in one flood or disaster with another. Therefore providing all messages at the onset of a disaster risk, including the CO hazard risks, is less likely to overload people in a stressful and busy situation with additional messages. Furthermore, it is difficult to find which actions are actually implemented at the local level in response to a warning or campaign at national level.

Other key interventions to prevent CO poisoning in floods include the promotion and provision of audible CO alarms, legislation to require CO detectors and mandate safety warnings on appliances which may emit CO and the establishment of surveillance systems for CO poisoning, with an enhanced system which can be activated in disaster situations. CO alarms must meet certain standards to be reliable and effective; in the United Kingdom this is the British (European) Standard BS EN 50291, which requires that alarms activate within 3 minutes of a threshold of 330ppm CO being crossed. Alarms meeting this standard were tested in a 2011 review of the effectiveness and long term reliability of CO alarms by the Health and Safety Executive (HSE), finding that such alarms are an effective back up precaution for properly installed and maintained combustion appliances.^30^

## Conclusions

The potential secondary consequences of any flood or disaster include the human health impact of CO. This paper describes the variety of circumstances in which CO poisoning may occur in flooding and highlights evidence suggesting poor public awareness of the wide variety of situations which may expose them to this risk in the period during and after a flood. This lack of awareness may be reflected in health care workers and is compounded by the problem of underreporting of cases.

CO poisoning is entirely preventable and thus an emphasis on planning for this predictable outcome of power outages is needed; raising awareness of CO poisoning must be part of disaster risk reduction plans.

This paper for the first time describes the different risks of CO poisoning posed by the different phases of a disaster. There is a specific need to recognise that any room in a building can harbour a CO emitting appliance in flooding; wood burners and rarely used chimney flues may become particularly problematic following a flood.

Finally, we call for training and awareness - raising throughout Europe using the CDC audience-tested CO toolkit as a model; the need to develop and evaluate culturally specific tools in appropriate languages must be explored by all countries.

## Recommendations

1. Public health workers and policy makers should consider establishing toolkits using the CDC toolkit approach, with plans in place to formally evaluate the acceptability and effectiveness of such interventions wherever possible. This represents a significant gap in research.
2. CO poisoning must form part of syndromic and event based surveillance systems for flooding and should be included in measures of the health impact of flooding.
3. CO monitors in the domestic environment should be sited not only in proximity to known CO emitters but also in locations where mobile or short term CO emitting appliances may be placed, including woodburners and infrequently used fireplaces.

## References

[1] World Health Organisation. Floods in the WHO European region: health effects and their prevention. Ed: Menne B, Murray V. (2013)

[2] Ahern M, Kovats S. The health impacts of floods. In: Few R, Matthies F, eds. Flood hazards and health: responding to present and future risks. London, Earthscan, 2006:28–53.

[3] Braubach M, Algoet A, Beaton M, Lauriou S, Heroux ME, Krzyzanowski M Mortality associated with exposure to Carbon Monoxide in WHO European Member States. Indoor Air. 2013; 23:115-125 10.1111/ina.12007 23025441

[4] Public Health England. Reducing the risk of carbon monoxide poisoning over winter (2013)

[5] Clarke S, Keshishian C, Murray V et al Screening for carbon monoxide exposure in selected patient groups attending rural and urban emergency departments in England: a prospective observational study BMJ Open 2012;2:e000877 10.1136/bmjopen-2012-000877 PMC3533103 23242237

[6] Wright J. Chronic and occult carbon monoxide poisoning: we don't know what we're missing. Emerg Med J 2002;19:386–90. 10.1136/emj.19.5.386 PMC1725950 12204981

[7] Centers for Disease Control and Prevention (CDC).Nonfatal, unintentional, non-fire-related carbon monoxide exposures---United States, 2004---2006. MMWR Morb Mortal Wkly Rep. 2008;57(33):896---899. 18716581

[8] Iqbal S, Law HZ, Clower JH, Yip FY, Elixhauser A. Hospital burden of unintentional carbon monoxide poisoning in the United States, 2007. American Journal of Emergency Medicine. 2012;30(5):657---664 10.1016/j.ajem.2011.03.003 21570230

[9] Hampson NB, Stock AL. Storm-related carbon monoxide poisonings: Lessons learned from recent epidemics. Undersea Hyperb Med. 2006;33(4): 257---263. 17004412

[10] Iqbal S, Clower JH, Hernandez SA et al. A review of disaster-related Carbon Monoxide Poisoning: Surveillance, Epidemiology, and Opportunities for Prevention. American Journal of Public Health 2012; 102(10): 1957-1963 10.2105/AJPH.2012.300674 PMC3490658 22897556

[11] Verrier A, Daoudi J, Gourier-Frry C, Salines Carbon monoxide poisoning surveillance: a French Environmental & Health surveillance system integrated in preventive policies G. Inj. Prev. 2010; 16(Suppl. 1): A263-A264.

[12] Euripidou E, Murray V. Public health impacts of floods and chemical contamination. J Public Health (Oxf). 2004 Dec;26(4):376-83. PubMed PMID:15598858. 10.1093/pubmed/fdh163 15598858

[13] Noji EK. The public health consequences of disasters. New York: Oxford University Press; 1997.

[14] Centers for Disease Prevention and Control (CDC). Carbon monoxide poisoning from hurricane associated use of portable generators --- Florida, 2004.MMWR Morb Mortal Wkly Rep. 2005;54(28):697---700. 16034315

[15] Cukor J, Restuccia M. Carbon monoxide poisoning during natural disasters: the Hurricane Rita experience. J Emerg Med. 2007 Oct;33(3):261-4. PubMed PMID:17976553. 10.1016/j.jemermed.2007.02.043 17976553

[16] Ochi S, Murray V, Hodgson S. The great East Japan earthquake disaster: a compilation of published literature on health needs and relief activities, march 2011-september 2012. PLoS Curr. 2013 May 13;5. PubMed PMID:23787732. 10.1371/currents.dis.771beae7d8f41c31cd91e765678c005d PMC3682758 23787732

[17] Hartling L, Brison RJ, Pickett W. Cluster of Unintentional Carbon Monoxide Poisonings Presenting to the Emergency Departments in Kingston, Ontario during ‘Ice Storm 98’ Canadian Journal of Public Health pp388-390 10.1007/BF03404080 PMC6990320 9926497

[18] Keshishian C, Sandle H, Meltzer M, Young Y, Ward R, Balasegaram S. Carbon monoxide from neighbouring restaurants: the need for an integrated multi-agency response. J Public Health (Oxf). 2012 Dec;34(4):477-82. PubMed PMID:22427702. 10.1093/pubmed/fds023 22427702

[19] McCann LJ, Close R, Staines L, Weaver M, Cutter G, Leonardi GS. Indoor Carbon Monoxide: A Case Study in England for Detection and Interventions to Reduce Population Exposure. Journal of Environmental and Public Health 2013; doi: dx.doi.org/10.1155/2013/735952 10.1155/2013/735952 PMC3649163 23690806

[20] Centers for Disease Control and Prevention (CDC). Unintentional carbon monoxide poisoning following a winter storm --- Washington, January 1993. MMWR Morb Mortal Wkly Rep. 1993;42(6):109---111 8429816

[21] Gasman JD, Varon J, Gardner JP. Revenge of the barbecue grill: carbon monoxide poisoning. West J Med. 1990;153:656---657. PMC1002654 2132567

[22] Daley WR, Smith A, Paz-Argandona E, Malilay J, McGeehin M. An outbreak of carbon monoxide poisoning after a major ice storm in Maine. J Emerg Med. 2000 Jan;18(1):87-93. PubMed PMID:10645845. 10.1016/s0736-4679(99)00184-5 10645845

[23] Wang SH, Emmerich SJ. Modeling the effects of outdoor gasoline powered generator use on indoor carbon monoxide exposures. NIST Technical Note 1637; August 2009:23. Available at: http://fire.nist.gov/ bfrlpubs/build09/PDF/b09009.pdf. Accessed June 1, 2011.

[24] BBC News. Flood death men overcome by fumes 2008 http://news.bbc.co.uk/1/hi/england/gloucestershire/7595243.stm Accessed 01/04/2014

[25] Public Health England/Environment Agency (PHE/EA): 2013 http://www.hpa.org.uk/webc/HPAwebFile/HPAweb_C/1317140405287 (Accessed on 10 January 2014)

[26] Pitt M. The Pitt review: learning lessons from the 2007 floods. http://www.cabinetoffice.gov.uk/thepittreview accessed 01/04/2014

[27] Kar-Purkayastha I; Finlay S; Murray V Low-level exposure to carbon monoxide BJGP. 2012 Volume 62, Issue 601, pages 404-404 ISSN: 0960-1643, Online ISSN: 1478-5242 DOI: http://dx.doi.org/10.3399/bjgp12X653480 10.3399/bjgp12X653480 PMC3404310 22867657

[28] Davies SD: Carbon Monoxide Poisoning: Recognise the symptoms and tackle the cause. Letter from the Chief Medical Officer (2013) Accessed 01/04/2014

[29] Rychetnik L, Frommer M, Hawe P, et al. Criteria for evaluating evidence on public health interventions. J Epidemiol Community Health 2002;56:119–27 10.1136/jech.56.2.119 PMC1732065 11812811

[30] Health and Safety Executive. Domestic Carbon Monoxide alarms (2011)

